# Microvascular dysfunction following deferred stenting strategy in ST-segment elevation myocardial infarction: a case report

**DOI:** 10.1093/ehjcr/ytad564

**Published:** 2023-11-14

**Authors:** Sebastian Albistur, Juan Torrado, Nicolás Niell, Rafael Mila

**Affiliations:** Department of Cardiology, Centro Cardiovascular Universitario—Hospital de Clínicas, Avda. Italia S/N, Montevideo 11600, Uruguay; Department of Internal Medicine, Jacobi Medical Center, Albert Einstein College of Medicine, Bronx, NY, USA; Department of Cardiology, Montefiore Medical Center, Albert Einstein College of Medicine, Bronx, NY, USA; Nuclear Medicine Department, Centro Uruguayo de Imagenología Molecular, Montevideo, Uruguay; Department of Cardiology, Centro Cardiovascular Universitario—Hospital de Clínicas, Avda. Italia S/N, Montevideo 11600, Uruguay

**Keywords:** ST-segment elevation myocardial infarction, Deferred stenting, Coronary physiology, Microcirculatory dysfunction, Case report

## Abstract

**Background:**

ST-segment elevation myocardial infarction (STEMI) has traditionally been managed with immediate reperfusion of the culprit artery, primarily through percutaneous coronary intervention and stent placement. Emerging data are highlighting the crucial importance of post-infarct microcirculatory function assessment.

**Case summary:**

This report presents a patient with an inferior STEMI who was successfully reperfused without stent implantation. Tools such as optical coherence tomography, fractional flow reserve, and positron emission tomography computed tomography N-13 ammonia were utilized, offering comprehensive insights into the anatomical and functional characteristics of both the epicardial vessel and microcirculation.

**Discussion:**

The recovery of the reversible component of microcirculatory dysfunction, observable as early as 5 days post-infarction, might carry significant implications for clinical decision-making. Such insights can potentially influence contemporary treatment strategies, including the consideration of deferred stenting. This case underscores the significance of post-infarct microcirculatory function and its potential impact on therapeutic approaches.

**Learning points**

- Post-infarction microcirculatory function: Post-myocardial infarction (MI), reversible disturbances in coronary microcirculation, such as distal embolization and vasospasm, coexist with irreversible myocardial necrosis. Understanding the kinetics of microcirculatory recovery, especially the reversible aspects, is crucial for functional assessment. Coronary flow capacity offers a more nuanced evaluation of microcirculation than coronary flow reserve, integrating data from invasive physiology and N-13 ammonia—cardiac PET imaging. Our case indicates reversible microcirculatory restoration within 5 days post-MI, a key consideration for deferred stenting strategies.
- Implications for deferred stenting: In STEMI management with deferred stenting, assessing the physiology of coronary lesions depends on a stable microcirculatory state. These assessments are crucial for determining the functional impact of the culprit lesion but are contingent on the microcirculation’s recovery dynamics. The role of physiological tools in lesion evaluation hinges on a comprehensive understanding of microcirculatory recovery patterns, which remains an area of emerging knowledge, particularly when comparing immediate to deferred stenting strategies.

## Introduction

Acute ST-elevation myocardial infarction (STEMI) typically results from an acute thrombotic occlusion of a coronary artery secondary to lipid-rich coronary atheromatous plaque complications.^1^ Timely primary angioplasty is the recommended treatment.^2^ However, questions remain regarding the optimal timing for stent placement.^3^ Stent placement may cause further microcirculation damage during deployment and carries risks such as stent thrombosis, intrastent restenosis, and bleeding complications.^4^ Lesions causing STEMI usually involve voluminous atheromatous plaques that result in non-flow-limiting stenoses.^5^ Thus, in select patients where effective mechanical reperfusion is achieved prior to stent implantation, there is an interest in exploring strategies that might mitigate the impact of stent placement on microcirculatory function.^6^ The DANAMI-3-DEFER study employed a deferred stenting strategy in selected cases.^7^

## Summary figure

**Figure:**
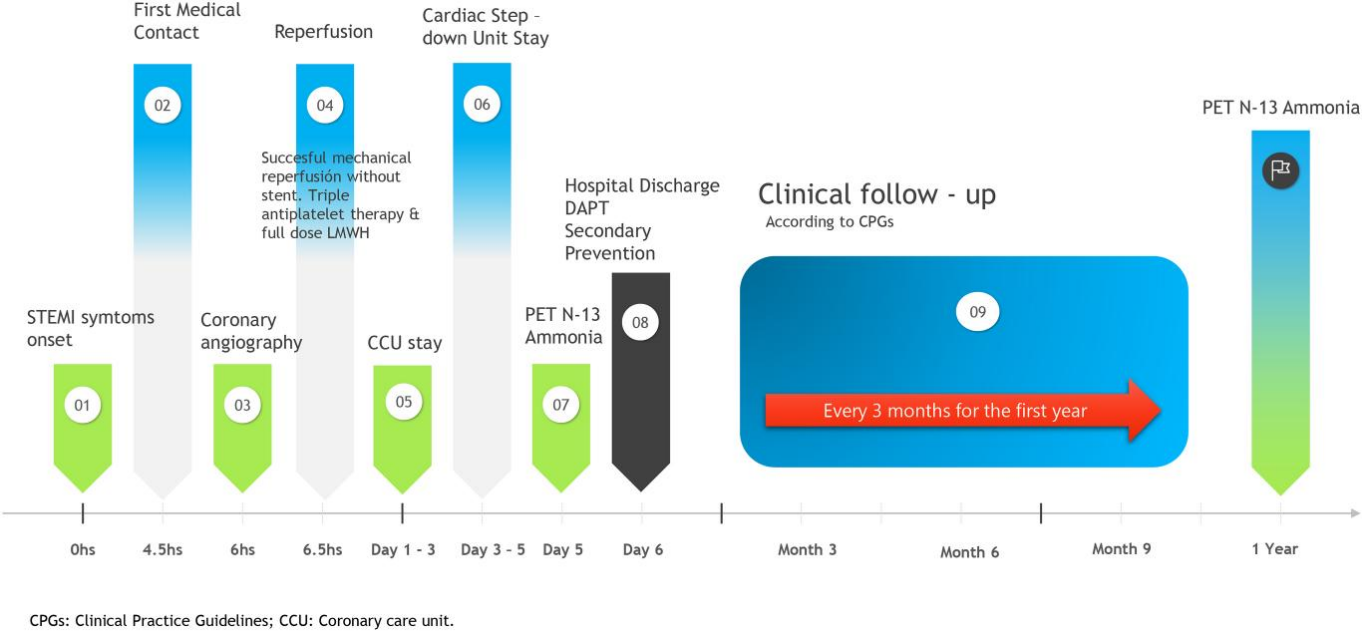


Coronary microvascular dysfunction (CMD) after primary angioplasty in infarct-related and non-culprit arteries is linked to increased long-term mortality.^8^ Coronary flow reserve (CFR) traditionally assessed microcirculatory function, but infarction-induced phenomena that reduce microcirculation’s ability to generate hyperaemia and changes in workload and resting flow in remote territories limit CFR’s utility. Coronary flow capacity (CFC) overcomes this limitation, integrating CFR with maximum hyperaemic flow, with positron emission tomography (PET) as the gold standard for quantification.^9,10^

We report a clinical case of a patient with inferior STEMI, who underwent successful myocardial reperfusion without stent implantation and experienced excellent outcomes. This case illustrates the pathophysiological reasoning behind the deferred stent strategy and highlights the potential role of coronary physiology on the 5th day post-infarction.

## Case report

A 74-year-old woman with a medical history of hypertension, treated with losartan 50 mg orally daily, presented to our hospital experiencing retrosternal chest pain at rest, persisting for 6 h. She has no history of diabetes or smoking and is not on any other medication. Further, she has no non-cardiac comorbidities and maintains a good functional status. Upon arrival, her vital signs were stable. A cardiovascular physical examination revealed regular heart rate and rhythm, no murmurs, rubs, or gallops, and peripheral pulses were palpable bilaterally without any oedema. The electrocardiogram confirmed a diagnosis of posteroinferior STEMI. Her laboratory results showed a haemoglobin of 13.2 g/dL (12.0–16.0 g/dL), white blood cell count of 6.2 × 10^9^/L (4.5–11.0 × 10^9^/L), and platelets of 240 × 10^9^/L (150–450 × 10^9^/L). The blood urea nitrogen was 15 mg/dL (7–20 mg/dL), creatinine was 0.9 mg/dL (0.6–1.2 mg/dL), and fasting glucose was 98 mg/dL (70–100 mg/dL), and high sensitivity troponin I level was 850 pg/mL (0–50 pg/mL).

**Figure 1:**
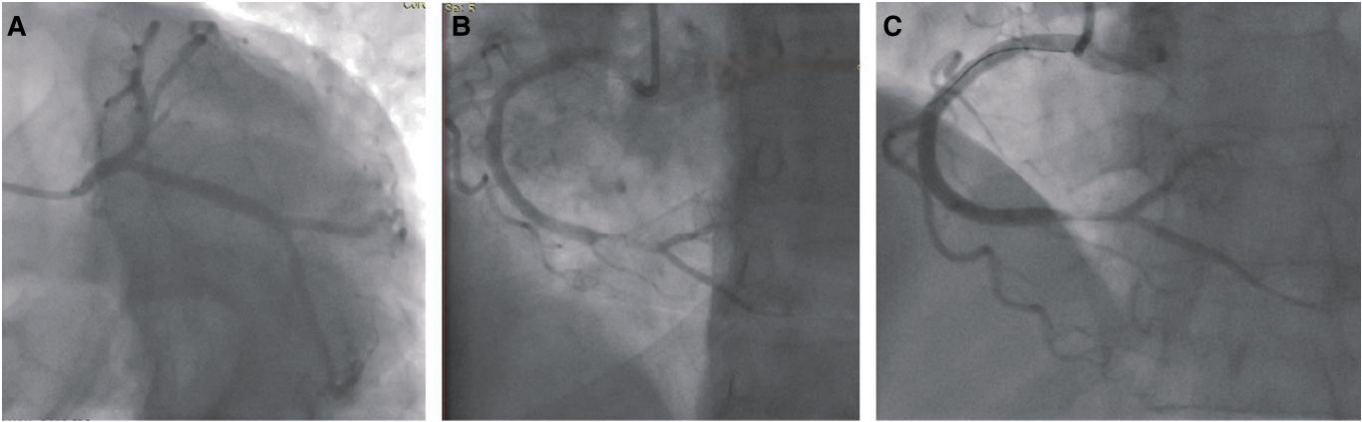
Initial coronary angiogram shows no obstructive coronary disease in the left main (LM), left anterior descending (LAD), and left circumflex (LCX) arteries (*A*). Image (*B*) shows a distal sub-occlusive obstruction in the distal right coronary artery (RCA). Image (*C*) shows partial resolution of the obstruction following thrombus aspiration resulting in flow restoration.

The patient underwent coronary angiography, which revealed a thrombotic sub-occlusive distal right coronary artery (RCA) stenosis (*Figure 1A* and *B*). The RCA was accessed with a Judkins Right 4 (JR4), and the lesion was crossed using a standard guidewire. Thrombus aspiration using an Export catheter (Medtronic AVE, Danvers) was performed, and significant red thrombotic material was obtained. Control injection showed a significant reduction in thrombus size, TIMI 3 flow (*Figure 1C*), and patient’s pain resolved with normalization of the ST elevation. In the context of a lesion with a high thrombotic burden, stemming from the thrombotic complication of an atheromatous plaque that extended distally and involved the bifurcation, and given that reperfusion was successful with no significant residual stenosis, we deferred stenting. The patient was transferred to the coronary care unit. During her treatment, the patient was administered intracoronary tirofiban with a continuous infusion over a 24 h period, accompanied by clopidogrel, acetylsalicylic acid, and enoxaparin.

**Figure 2:**
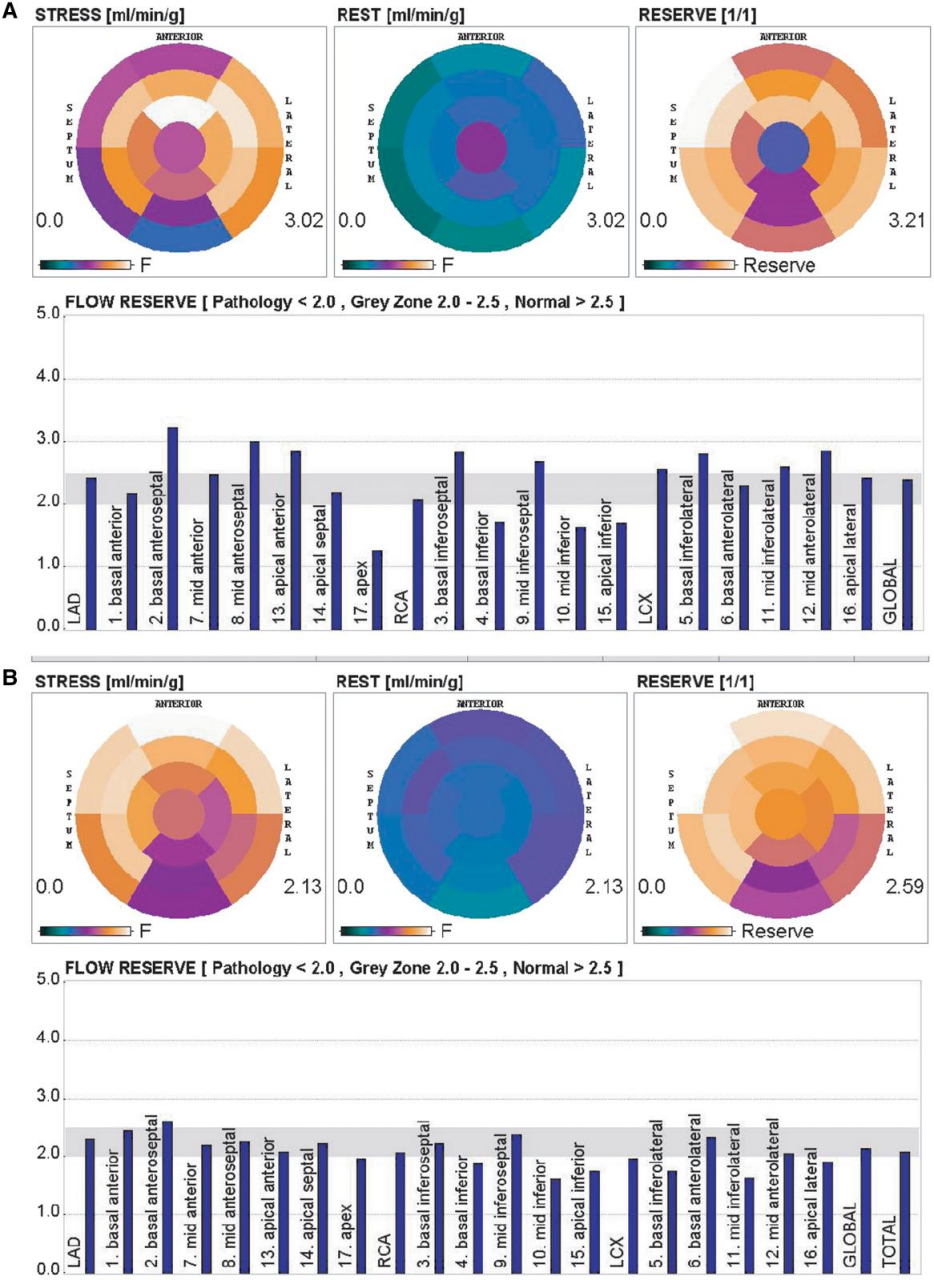
PET CT perfusion study demonstrating segmentation performed based on the 17-segment model. (*A*) Five days after STEMI. The culprit vessel myocardial region corresponds to segments 4, 10, 15, and 17, while the remaining segments are classified as non-culprit vessel myocardial regions. (*B*) One year after STEMI. This image reveals persistent abnormalities in stress flow and coronary flow reserve within the culprit vessel region.

On the 5th day, a diagnostic 13 N-ammonia myocardial perfusion imaging using a positron emission tomography computed tomography (PET CT) scanner was performed to assess the CFR and CFC as described by Cho *et al*.^11^ The CFR was found to be abnormally reduced in the basal, mid, and apical inferior segments, as well as in the apex (*Figure 2A*). Accordingly, we identified a culprit vessel region based on the affected segments, which was then compared to the non-culprit vessel myocardial region.

**Figure 3:**
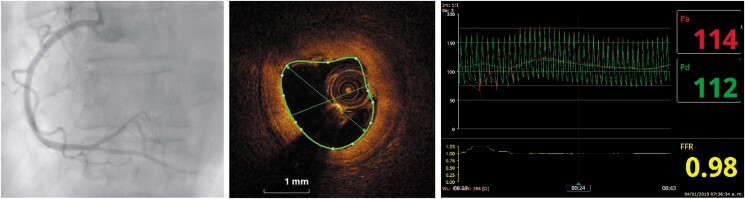
(*A*) Angiography 5 days after STEMI showing almost complete resolution of obstruction. (*B*) OCT image allows visualization of non-obstructive fibrolipidic plaque and remaining thrombus due to complication of the atheroma plaque. (*C*) FFR of 0.98 and hyperaemic distal mean arterial pressure of 112 mmHg.

A subsequent coronary angiogram was performed with the aid of optical coherence tomography (OCT) and at the 5th day post-MI. No significant angiographic residual stenosis was observed (*Figure 3A*). The OCT imaging demonstrated a complicated fibrolipidic atheroma plaque located at the distal RCA. The remaining thrombus and atheroma plaque resulted in a coronary stenosis with a minimum luminal area of 3.15 mm² (*Figure 3B*). To further assess the haemodynamics of the coronary arteries, a pressure wire was utilized to obtain the fractional flow reserve (FFR = 0.98) (*Figure 3C*).

Using haemodynamic data from catheterization and PET, we calculated coronary resistance. To estimate the distal pressure value, we applied the FFR value to non-invasive pressure quantification obtained during maximal hyperaemia under dipyridamole administration (78 ∗ 0.98 = 76 mmHg). The flow used was the average of affected segments (CFR < 2) during maximal hyperaemia (1.575 mL/min/g). Therefore, the resistance offered by the coronary circulation per gram of tissue during maximal hyperaemia was 48.25 mmHg/(mL/min/g). For the calculation of coronary resistance in the left anterior descending artery (LAD) and left circumflex artery (LCx), we did not use FFR to correct the mean pressure value during maximal hyperaemia. We obtained a coronary resistance value of 35.94 mmHg/(mL/min/g) and 30.83 mmHg/(mL/min/g), respectively.

One year later, a repeat 13 N-ammonia PET CT study was performed to re-evaluate myocardial perfusion. We obtained a mean pressure value of 60 mmHg during maximal hyperaemia. The resistance in the infarcted sector was 51.23 mmHg/(mL/min/g) compared to 32.79 mmHg/(mL/min/g) and 37.29 mmHg/(mL/min/g) in the LAD and LCx, respectively (*Figure 2B*).

Comparing the magnitude of coronary resistance in the affected segments as a percentage of the average resistance in unaffected vessels (i.e. LAD and LCx), we obtained a value that was 44.5% higher in the affected segments 5 days after the infarct and 46.2% higher after one year.

**Figure 4:**
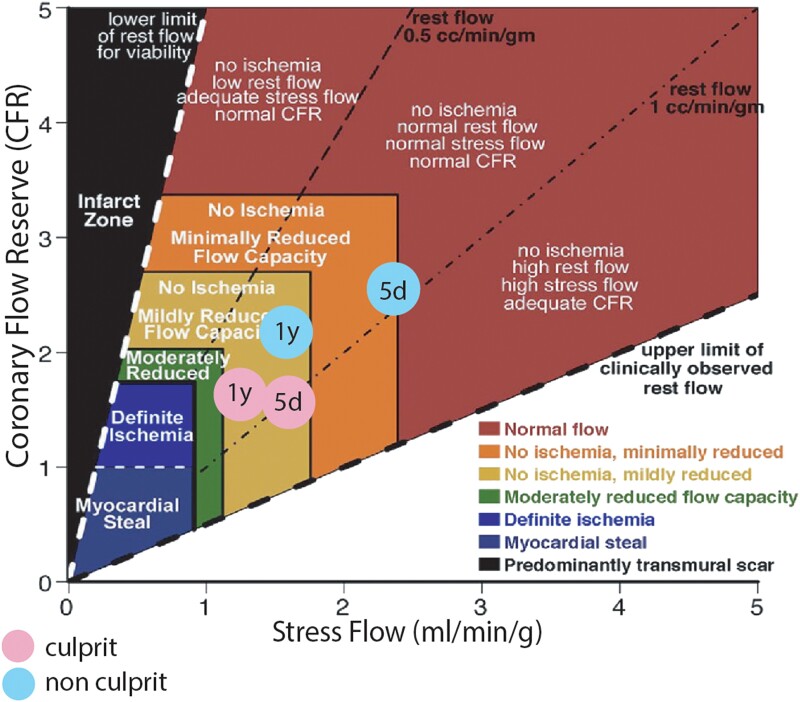
Shows CFC of culprit and non-culprit vessel region at 5 days and 1 year after successful reperfusion of STEMI. 5d, 5 days after STEMI; 1y, 1 year after STEMI.

Based on CFR and maximum stress flow data, we constructed a CFC diagram.^12^ This diagram displays both the affected and not affected myocardial segments by the infarct. *Figure 4* represents the CFC in the affected (culprit) and non-affected (non-culprit) territories at 5 days and 1 year after STEMI.

The patient had an excellent clinical evolution, remaining symptom-free at 1 year follow-up.

The data supporting this case report are available as supplementary material accompanying this manuscript.

## Discussion

Anatomical variations in coronary arteries can render stenting less optimal. In situations involving notable diameter discrepancies or bifurcations, intricate stenting techniques heighten the risks of side branch occlusion, stent thrombosis, and restenosis.^4^ In this instance, the lesion in the RCA was distally positioned, with its bifurcation affected by thrombus, leading us to contemplate a deferred stenting strategy. Successful mechanical reperfusion was achieved without stenting. Evidence supports deferring stent implantation in safe select patients.^7^ The choice of a 5-day period for a new haemodynamic study was based on data suggesting microcirculatory function recovery within that time.^13–15^ Microcirculatory dysfunction in STEMI-affected regions has irreversible (myocardial necrosis) and reversible components due to dynamic microcirculatory phenomena, embolic events, and inflammatory mediators.^9^ If reversible microcirculatory dysfunction resolves significantly by Day 5 post-MI, FFR would be valid for assessing residual stenosis.

Follow-up angiography showed antiplatelet and anticoagulant therapy significantly reduced luminal obstruction, which was nearly imperceptible (*Figure 3*). Optical coherence tomography identified the plaque complication site, corresponding to a fibrolipidic atheroma plaque causing mild to moderate stenosis with residual thrombosis.

The pressure guidewire was employed to measure the distal pressure during maximal hyperaemia, providing insights into both the coronary microcirculation status and the haemodynamic significance of the residual stenosis. In mechanical reperfused STEMI without stenting, obstruction geometry varies due to thrombus presence, which tends to resolve over time. Residual stenosis is often moderate. On Day 5, if reversible microcirculatory dysfunction improves, pressure guidewire information might be valid for decision-making; however, this notion is far from being conclusively proven.^9^

Considering angiographic, intravascular anatomical, and haemodynamic findings, the procedure was completed without stenting. The patient remained symptom-free at 1-year follow-up. A PET CT myocardial perfusion study showed persistent microcirculatory dysfunction in the culprit territory like Day 5 post-infarction. The diagram in *Figure 4* shows a mild to moderate CFC decrease, corresponding to a non-ischaemic zone. The microcirculatory dysfunction caused a 45% increase in coronary resistance in the infarcted territory, suggesting predominant irreversible dysfunction due to myocardial necrosis.

**Figure 5:**
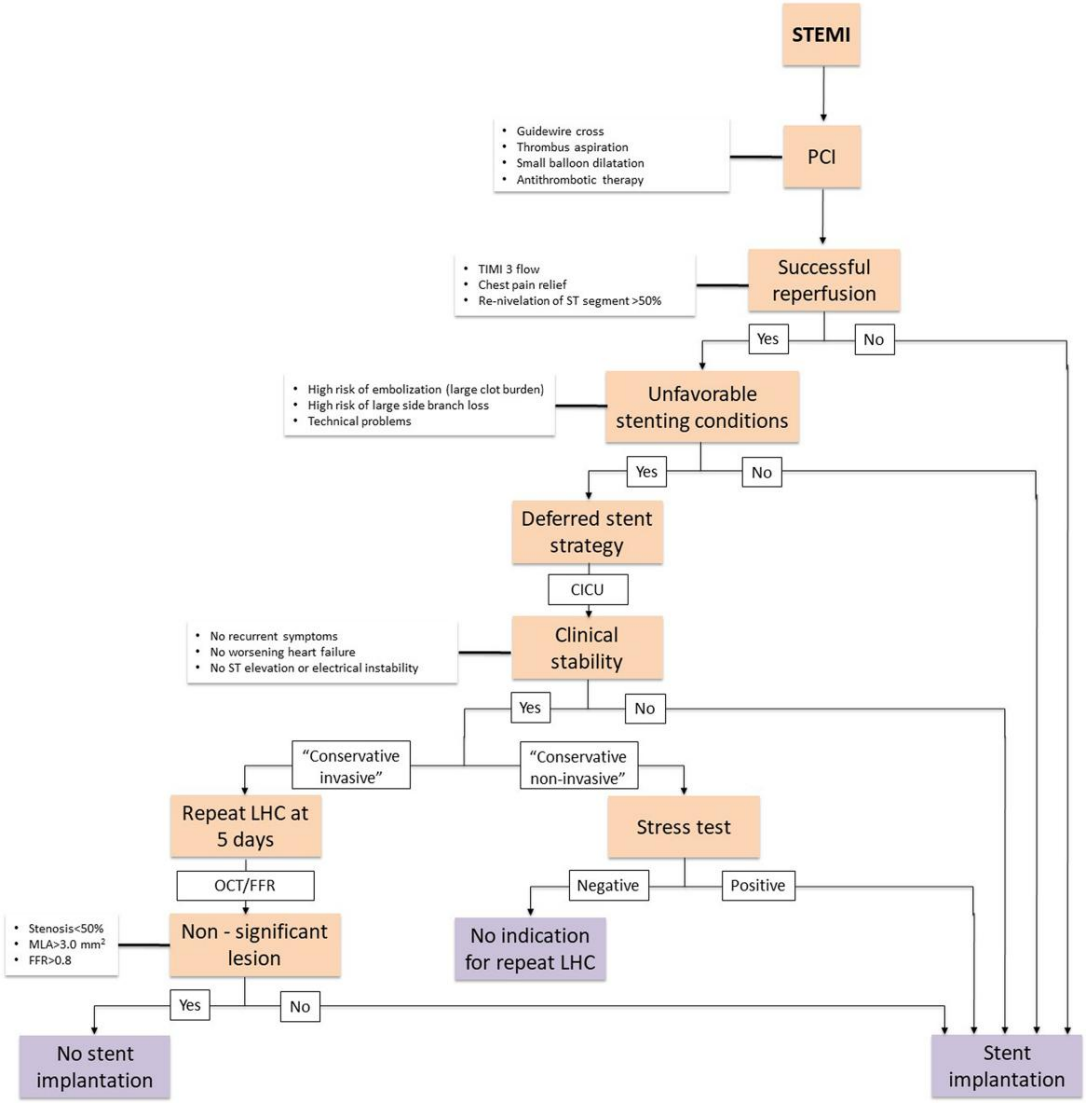
STEMI PCI alternative algorithm. LHC, left heart catheterization; CICU, cardiovascular intensive care unit.

The recuperation of microcirculatory function post-infarction is pivotal. Assessing microcirculation in STEMI can inform the strategy for stent implantation, particularly in lesions with a high thrombus burden identified as the culprit.^16^ Coronary physiology harbours multiple tools for the evaluation of ischaemic CMD. Should significant recovery be evidenced by the 5th day, this might reinforce the utilization of these coronary physiology tools, particularly when contemplating deferred stenting strategies.^17^ Given the current uncertainty surrounding this recovery kinetics, we introduce a speculative algorithm with the intent of sparking discussion on how understanding microcirculatory function might potentially influence clinical decision-making (*Figure 5*).

## Supplementary Material

ytad564_Supplementary_Data

## Data Availability

The data underlying this article are available in the article and in its online supplementary material.

## Lead author biography

Dr Rafael Mila is an Assistant Professor of Cardiology at the University Cardiovascular Center—Hospital de Clínicas. He completed his training as an interventional cardiologist at the Hospital Clínico San Carlos in Madrid, Spain. His area of expertise is coronary physiology. Dr Mila has been involved in this field since the beginning of his medical career, working in the departments of Biophysics and Pathophysiology at the Faculty of Medicine, Universidad de la República, Montevideo, Uruguay.

## Supplementary material

**Consent:** The patient (or their legal representative) has provided informed consent for the publication of this clinical case, including any accompanying images and anonymized details, in this scientific journal. The patient has been informed about the purpose of the publication and understands that their identity will be protected.

We hereby confirm that informed consent was obtained from the patient for the publication of this case report and any associated images. All procedures and the reporting of this case are in compliance with the guidelines set forth by the Committee on Publication Ethics (COPE).

**Funding:** The optical coherence tomography (OCT) and fractional flow reserve (FFR) procedures were funded by research grants from the National Agency for Research and Innovation (Agencia Nacional de Investigación e Innovación, ANII) and the Program for the Strengthening of Scientific Research (Programa de Fortalecimiento a la Investigación Científica, PROFIC), both of which are government agencies in Uruguay. The perfusion studies conducted with PET were funded by the Uruguayan Center for Molecular Imaging (Centro Uruguayo de Imagenología Molecular, CUDIM), also a government-funded institution.
